# An Unusual Relation in an Infant With Left Hemitruncus and Tetralogy of Fallot Along With Pulmonary Valve Syndrome: A Case Report

**DOI:** 10.7759/cureus.62916

**Published:** 2024-06-22

**Authors:** Benumadhab Ghosh, Isha Sahai, Vaibhav Raut, Vaibhav Mahalle, Gajendra Agrawal

**Affiliations:** 1 Cardiology, Jawaharlal Nehru Medical College, Datta Meghe Institute of Higher Education and Research, Wardha, IND

**Keywords:** pulmonary stenosis, digeorge, ventricular septal defect, tetralogy of fallot, infections, hemitruncus, left pulmonary artery

## Abstract

The abnormal origin of the left pulmonary artery (LPA) from the ascending aorta is a rare cardiac condition that is often associated with several other congenital defects. In this paper, we report the case of an infant who presented with recurrent infections and was prenatally diagnosed with tetralogy of Fallot (TOF). During echocardiography, various other cardiac defects such as ventricular septal defects (VSD), pulmonary stenosis (PS), and dilated right heart chambers were identified. Furthermore, cardiac catheterization revealed an anomalous origin of the LPA arising from the aorta associated with a narrow pulmonary annulus. Due to both conditions sharing a similar embryological course, the condition is commonly associated with a conotruncal defect known as DiGeorge syndrome. Together, the overall combination of cardiac anomalies is both unusual and unique. This case study explains the clinical associations, embryological origin, and surgical management of this condition in an infant.

## Introduction

Hemitruncus is a rare genetic abnormality in which a single pulmonary artery develops from the ascending aorta. According to research, about 1% of people have some form of aberrant pulmonary artery origin from the ascending aorta. The aorta can have an aberrant origin of the left or right pulmonary artery (AOLPA), with the left pulmonary artery (LPA) accounting for about 0.03% of congenital cardiac manifestations. It is thus a much rarer condition [1,2], which is chiefly associated with a congenital heart condition known as the tetralogy of Fallot (TOF). TOF is a cyanotic condition characterized by ventricular septal defect (VSD), overriding of the aorta, stenosis of the pulmonary valve (PS), and hypertrophy of the right ventricle [3].

There are various other conditions associated with the anomalous origin of the LPA. About 7.1% of cases have been found to be associated with DiGeorge syndrome, a genetic condition involving 22q11 gene deletion [4]. The incidence of this syndrome is observed to be around one in 4,000 live births [5]. In DiGeorge syndrome, the annulus of the pulmonary valve is primarily hypoplastic in nature, along with a stenotic and dysplastic pulmonary valve. The VSD is usually located in the peri-membranous septum, although there can be an extension of the defect to the muscular septum. Rarely, multiple muscular VSDs may be present [6].

In this report, the authors present a case of an infant who presented with recurrent infections and was diagnosed with an anomalous origin of LPA. The report explains the detailed management of the patient.

## Case presentation

An infant boy was brought to a tertiary hospital in central India by his parents due to recurrent infections since birth. The child had a known case of tetralogy of Fallot (TOF), as diagnosed by echocardiography in our hospital. It had been advised that the patient undergo cardiac surgery, but during the procedure, LPA arising from the ascending aorta was found. Due to doubtful operability, a further catheterization study was recommended, and the patient came in for further investigations.

The patient has a history of recurrent colds and coughs. There was no record of associated fever, distress, or desaturation. Family history is insignificant; the child is a product of a non-consanguineous marriage. The neonate was 2.5 kilograms at the time of birth, was delivered by standard vaginal delivery, and stayed in the neonatal intensive care unit (NICU) for seven days. The baby did not cry immediately after birth. He has been immunized per the Indian Association of Paediatrics (IAP) schedule and has developed milestones for his age. Anthropometric analysis shows the following measurements, as shown in Table 1.

**Table 1:** Anthropometric measurements of the infant The table is the sole creation of the authors. SD: standard deviation

Parameters	Observed	Expected	Percentage and deviation	Inference
Length for age	65 cm	75 cm	-3 SD	Severe chronic stunting
Weight for length	6.1 kg	7.5 kg	-2 SD to -3 SD	Moderate wasting
Weight for age	6.5 kg	9.1 kg	66.6%	Grade 2 malnutrition

**Figure 1:**
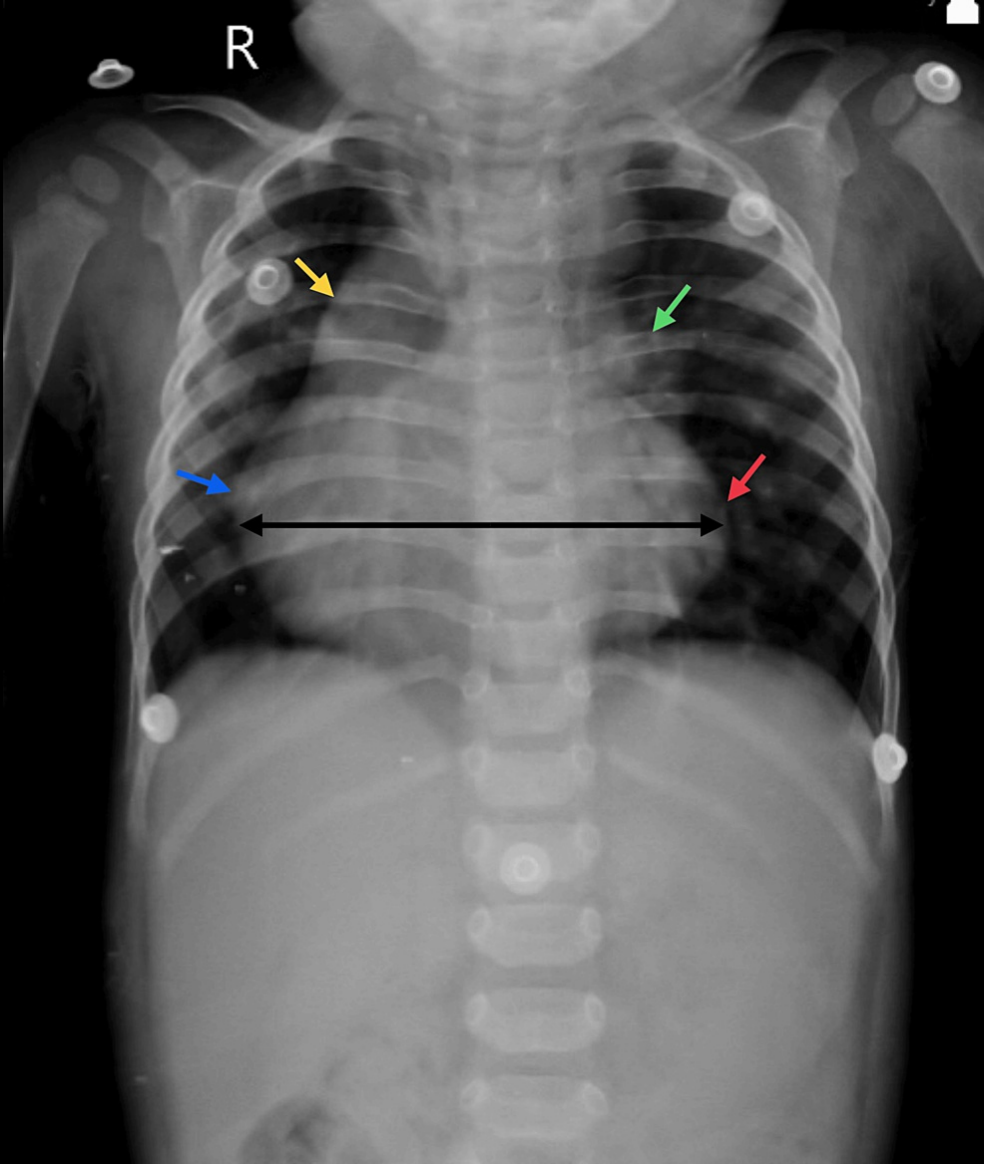
Chest X-ray showing RA enlargement (marked by the yellow arrow), RV enlargement (marked by the blue arrow), RV apex (marked by the red arrow), and hump suggestive of AOLPA (marked by the green arrow) The image was taken by the authors. R: right atrium, RV: right ventricle, AOLPA: anomalous origin of left pulmonary artery

During the general examination, the patient was conscious and comfortably lying on the bed. His heart rate was measured at 112 per minute, while his respiratory rate was 48 per minute. He was afebrile, with a blood pressure of 85/40 mm hg (millimeters per mercury), normal jugular venous pressure, and pulse oximetry (SpO_2_ 82%). There was no sign of cyanosis or jaundice. On clinical examination of the patient, a pan systolic murmur (grade four) was heard at S1 and S2. Air entry was bilaterally equal, and no subcostal retractions were seen. On laboratory analysis, his hemoglobin was found to be 12.1 g/dl. The following was observed on a chest X-ray, posterior-anterior view: situs solitus, mesocardia, RA enlargement, RV apex, a prominent hump on the left side suggestive of AOLPA, and a decreased Qp (pulmonary blood flow) in the right lung, as shown in Figure 1.

A diagnostic catheterization was performed to assess the branch of the pulmonary artery, including its size, origin, and operability, as shown in Table 2. Access was gained through the right femoral artery (RFA) and vein (RFV), using 4F- and 5F-sized sheaths. Three ml/kg of contrast was used, and no complications were associated during the procedure. Calculations were made for the pulmonary and systemic blood flow, and the findings are presented in Table 3. A high Qp/Qs ratio (more than two) suggests a left-to-right shunt and an immediate need for surgery.

**Table 2:** Catheterization lab measures of pressure and saturation in various cardiac structures The table is the sole creation of the authors. LA: left atrium, RV: right ventricle, PA: pulmonary artery, SVC: superior vena cava, AAO: ascending aorta, LV: left ventricle, LPA: left pulmonary artery

Site	Pressure	Saturation
RA	10	-
LA	10	-
RV	75	80%
PA	30/11	-
SVC		69.8%
AAO	70/41	94%
LV	75/8	98%
LPA	66/42	88%

**Table 3:** Calculations for blood flow in the left and right lungs The table is the sole creation of the authors. Qp: pulmonary blood flow, Qs: systemic blood flow, PVRI: peripheral vascular resistance index, SVRI: systemic vascular resistance index

Calculations	Left lung	Right lung
Qp	9.3 L/m	5.2 L/m
Qs	4.1 L/m	5.1 L/m
Qp/Qs	2.23:1	1.19:1
PVRI	3.98	2.41
SVRI	11.6	11.26
PVRI/SVRI	0.3	0.21

**Figure 2:**
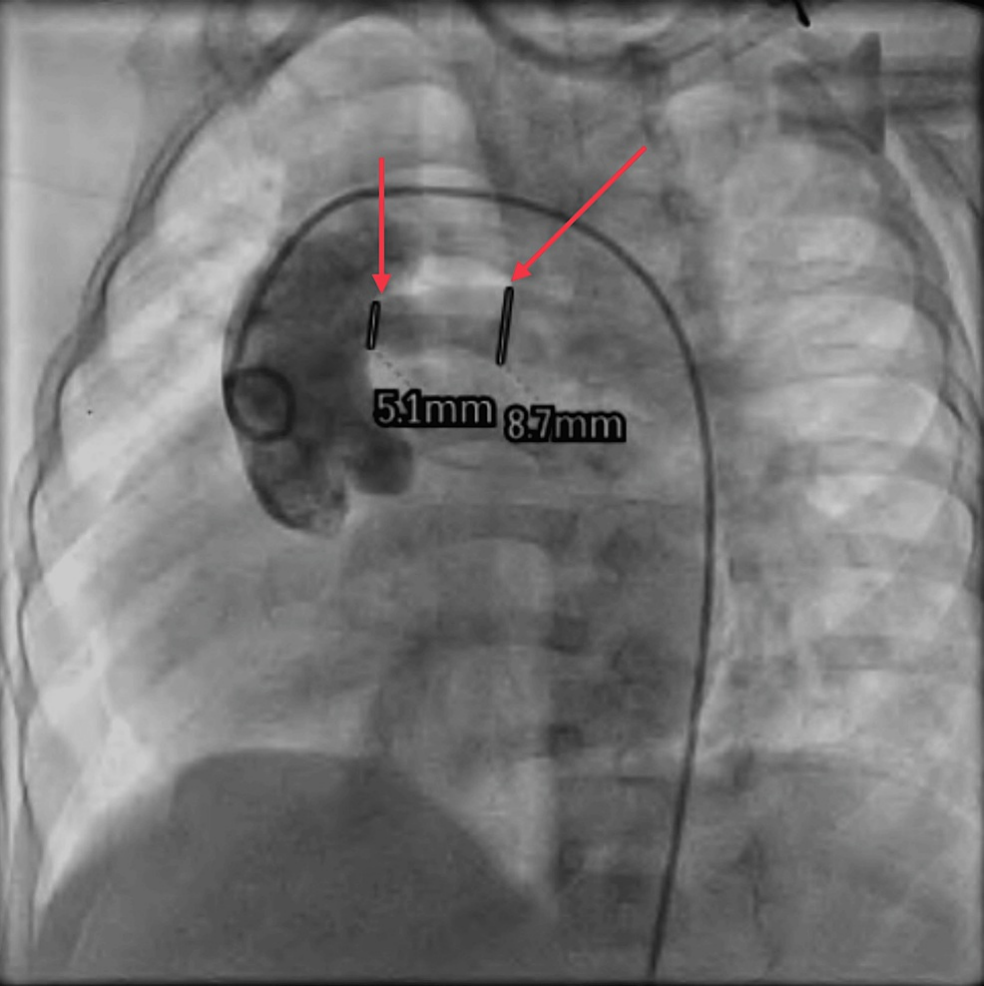
Angiogram showing the left aortic arch with an AOLPA from the ascending aorta (marked by the red arrows) The image was taken by the authors. AOLPA: anomalous origin left pulmonary artery

**Figure 3:**
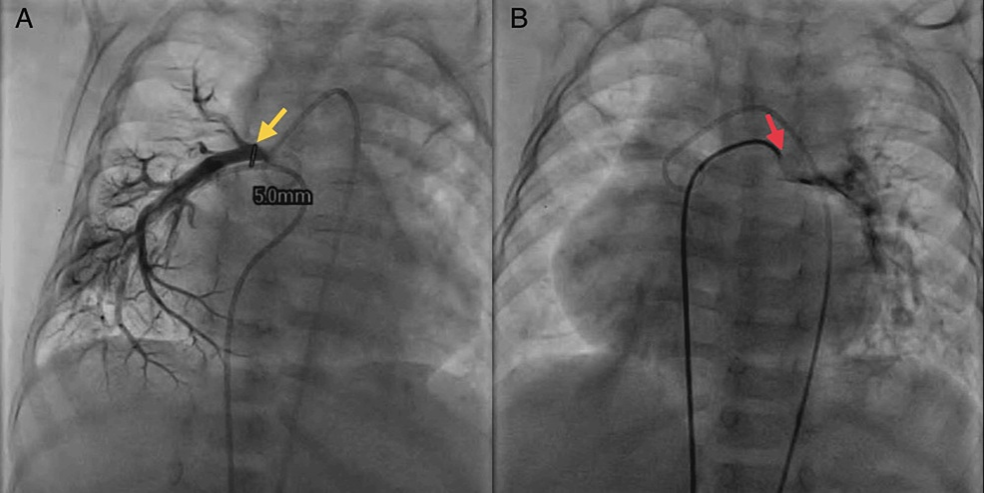
CT angiogram showing (A) LPA measuring 5 mm in size and (B) catheter inserted through IVC to RA to RV to LV to aorta to LPA The image was taken by the authors. IVC: inferior vena cava, RA: right atrium, RV: right ventricle, LV: left ventricle, LPA: left pulmonary artery

A flush angiogram was also performed via RFA to the aorta, which showed a left aortic arch with a regular branching pattern of neck vessels, as shown in Figure 2. AOLPA from the ascending aorta with mild narrowing at the origin and good distal arborization of the left lung field and levo-phase showed a pulmonary venous return to the left atrium. Angiography revealed the size of the LPA, which was about 5 mm in diameter at the base, as shown in Figure 3A. The catheter was inserted from the inferior vena cava to the right ventricle via the right atrium, then to the aorta via the left ventricle, and then to the LPA, as shown in Figure 3B.

**Figure 4:**
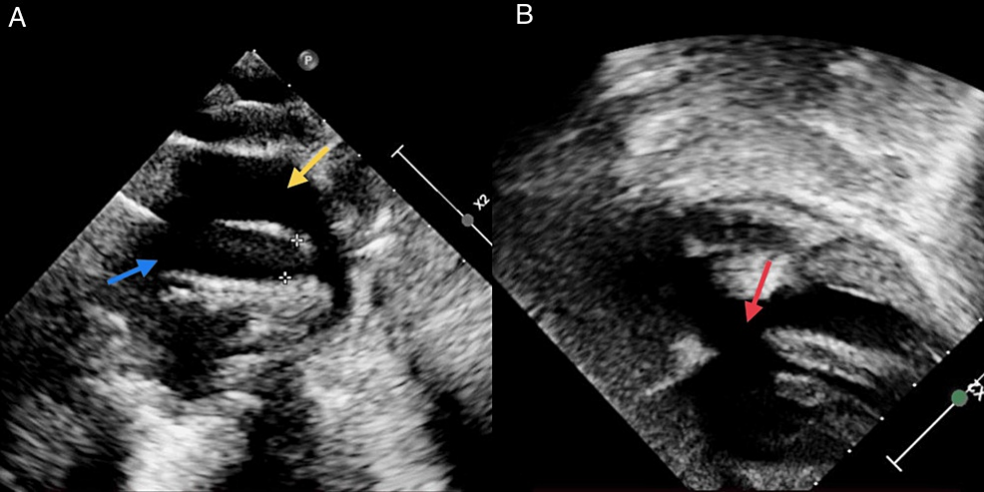
Echocardiography images showing (A) the left pulmonary artery (marked yellow) and ascending aorta (marked blue) and (B) showing VSD (marked red) The image was taken by the authors. VSD: ventricular septal defect

On echocardiography, TOF was indicated by a large subaortic VSD and severe subvalvular and valvular pulmonary stenosis. In addition, a 10 mm LPA arising from the aortic arch, a 5 mm pulmonary annulus, and a 6 mm RPA were seen. The right atrium and right ventricles seemed to be dilated. A 3 mm patent ductus arteriosus was present with mild aortic regurgitation, as shown in Figures 4A-4B.

A pre-catheterization diagnosis was made for the presence of TAF, large non-restrictive subaortic VSD, aortic overriding, severe valvar and infundibular pulmonary stenosis with a non-confluent branch of PA, the main pulmonary artery (MPA) giving rise to right pulmonary artery (RPA), and the left aortic arch giving rise to AOLPA. 

A team of experts including interventional cardiologists, cardiovascular surgeons, radiologists, technicians, and pediatric surgeons decided on the treatment plan for the patient. This involved intrinsic cardiac repair via a transannular patch repair made for VSD closure. A right ventricle-to-pulmonary artery conduit with reimplantation of LPA to the MPA or conduit was also done.

The infant was kept in the pediatric intensive care unit (PICU) for a while for monitoring; his vitals were normal, and there were no postoperative complications. Post-surgery follow-up of the infant was done. He was vitally stable without any complications and was kept under observation in the PICU. Several parameters, which included oxygen saturation, pulse, blood pressure, physical appearance, and physical movement, were observed and noted. All of them were satisfactory and normal. The patient was feeding well and was discharged after 10 days.

## Discussion

An aberrancy could be seen in the fifth aortic arch during embryogenesis, a feature that can persist as an anomalous LPA from the ascending aorta. DiGeorge syndrome follows the same pathway during embryogenesis, arising from the arch of the aorta, and thus presents as an associated genetic cardiac condition [7]. On the left side, the sixth aortic arch, originating from the post branchial plexus, originates to the LPA during the eighth week of development [8].

According to an article by Loomba et al., out of 113 patients with anomalous LPA arising from the ascending aorta, the most common associated cardiac abnormality was TOF, which was seen in 59 cases (52.2% of the total) [9,10]. In a study of 216 patients diagnosed with TOF, it was noted that 38.9% of them had pulmonary artery abnormalities, with isolated LPA stenosis being the most common [11].

Associated cardiovascular abnormalities define the presentation in around 40% of patients with TOF. Manifestations include patent ductus arteriosus (PDA), supravalvular pulmonary stenosis, atrial septum defect (ASD), stenosis in pulmonary artery branches, supravalvular pulmonary stenosis, atresia of the pulmonary valve, and hypoplasia of pulmonary artery branches, which might form at the fetal life as there is a progression of the sub-pulmonary infundibular contraction. Along with this, there can also be a disconnected left pulmonary artery originating from the ascending aorta (formerly known as hemitruncus), the ductus arteriosus giving rise to an LPA, absence of the LPA and pulmonary valve, aberrant coronary arteries, aberrant pulmonary venous return, atrio-ventricular septum defect (AVSD), double-outlet right ventricle, and incompetence of aorta [12].

Various anomalies that can be found in association with anomalous LPA origin may include an aberrant right subclavian artery, multiple aortopulmonary collateral vessels from the right lung, VSD, and a double-outlet right ventricle due to persistent left fifth aortic arch extending from the innominate vein to the ductus arteriosus [13,14].

Assessing the anatomical sites of the aortic arch in routine prenatal screening through a three-vessel and trachea (3VT) view can help physicians in the early diagnosis of pulmonary arterial anomalies. Thus, it may improve morbidity among these patients [15]. Pulmonary over-circulation causes pulmonary hypertension, which can eventually progress to heart failure. AOLPA leads to increased pulmonary circulation due to a decrease in the resistance of pulmonary vessels and thus be a further cause of heart failure [16]. It has been documented that surgical correction in such patients significantly improves survival in a prenatally detected case using fetal echo [17]; in our patient, we performed surgery. This surgery was performed using a conduit between the right ventricle and the pulmonary artery, followed by an LPA implantation using the annular patch.

## Conclusions

LPA originating from an anomalous site of the ascending aorta, also known as hemitruncus, is a rare condition. It is usually associated with a variety of cardiac manifestations, which may include DiGeorge syndrome, VSD, multiple major aorta pulmonary collateral arteries, double-outlet right ventricle, an aberrant subclavian artery, and hypoplastic pulmonary valve syndrome.

We present a case of aberrant origin of LPA from the ascending aorta, which was associated with TOF and a hypoplastic pulmonary valve, in an infant who presented with complaints of recurrent infections. These cardiac manifestations are rare in association, making them unique. The patient was managed surgically by intrinsic cardiac repair with a transannular patch repair performed for VSD closure. A right ventricle-to-pulmonary artery conduit with reimplantation of the LPA to the MPA or conduit was also conducted. The infant was kept in the PICU, the vitals were normal, and there were no post-op complications. On follow-up, the patient had stable vitals, oxygen saturation, pulse, blood pressure, physical appearance, and physical movement were normal. The patient was feeding well and had an excellent post-op prognosis.
